# Temperature-Mediated Gel Texture Transformation in Starch Noodles: In Respect of Glass Transition Temperature T_g_’

**DOI:** 10.3390/gels11080639

**Published:** 2025-08-13

**Authors:** Hongxiao Liu, Qing Hu, Sha Yang, Lina Liu, Xuyan Dong

**Affiliations:** 1 College of Food Science and Engineering, Qingdao Agricultural University, Qingdao 266109, China; liuhongxiao0621@163.com (H.L.); hqingu@163.com (Q.H.); liuln1023@163.com (L.L.); 2 Key Laboratory of Novel Food Resources Processing, Ministry of Agriculture and Rural Affairs, Key Laboratory of Agro-Products Processing Technology of Shandong Province, Institute of Food & Nutrition Science and Technology, Shandong Academy of Agricultural Sciences, 23788 Gongye North Road, Jinan 250100, China

**Keywords:** starch noodles, texture, enzymatic hydrolysis, scanning electron microscopy

## Abstract

Potato starch noodles (PSN), a characteristic gluten-free Asian food, are essentially high-concentration starch gels (about 35% starch) formed through gelatinization and retrogradation. This study systematically investigates freezing temperature effects, particularly across the glass transition temperature, on PSN texture and microstructure. We found that fresh PSN have a freezing point of −1 °C, supercooling temperature of −4.5 °C, and a T_g_’ value of −3.1 °C. Freezing significantly reduced the adhesiveness of PSN and increased the hardness. During the 48 h freezing process, noodles frozen at −3 °C, the closest to T_g_’, exhibited the highest hardness (14,065.77 g), springiness (0.98), cohesiveness (0.93), chewiness (11,971.06), and resilience (0.84), and the least adhesiveness. PSN frozen within the range near T_g_’ (−3 °C) showed superior texture, continuous solid cross-section, and dense surface, attributed to the reverse transformation of starch, high mobility of starch chains, and smaller ice crystals. PSN frozen at −3 °C for 24 h displayed the most compact and desirable texture compared to the other samples. These findings deepen the understanding of the role of glass transition temperature in the texture formation of starch gel during freezing and provide valuable insights for optimizing the frozen processing of starch gel-based food.

## 1. Introduction

Starch noodles are made from pure plant starch (such as potato, sweet potato, mung bean, etc.) as raw materials and are a major category of Asian noodles [1]. Unlike wheat flour-based noodles, the unique raw material selection of starch noodles makes it ideal for a gluten-free diet. Compared to sweet potato starch noodles, those made from potato starch generally have a smoother surface, a more uniform and shiny appearance, a more delicate and smooth taste, and lower breakage and paste rates [2]. The production process begins with a pregelatinized starch binder paste, followed by six steps including forming the starch dough with mixed starch powder and water, extruding the dough into fine noodles, cooking, freezing, thawing, and packaging [3]. Among these, freezing plays a critical role in determining the final texture. It transforms the noodles from a sticky paste into a non-sticky, springy, and tough retrograded gel, effectively reducing adhesiveness between strands and forming their characteristic edible quality [4].

However, significant controversy exists regarding optimal freezing temperatures (reported from −40 °C to 0 °C) and durations. Yang et al. [5] prepared potato starch noodles (PSN) at 0 °C for 36 h and obtained a desirable texture. Fan et al. [6] reported that starch noodles frozen at −2 °C to −8 °C for 10 h, followed by −18 °C for 6 h, had the best texture; longer freezing times reduced cooking and shear resistance. They proposed that water extruded as ice during freezing forms an “ice coat” around the noodles, greatly reducing adhesiveness and improving toughness. Sun et al. [7] found that the freezing temperature of −40 °C would destroy the internal structure of starch gel and reduce the gel strength of starch noodles. Thus, it could be concluded that starch noodles frozen for 6 h achieved higher tensile strength than those frozen for 3 or 24 h. Nguyen et al. [8] evaluated the edible quality and resistant starch content of starch noodles affected by different freezing temperatures (−5, −10, −15, and −20 °C), suggesting that freezing at −10 °C for 18 h is recommended to produce mung bean starch noodles with high resistant starch content and good quality. Zeng et al. [9] found that the maximum retrogradation and toughness of starch noodles occurred at −2 °C. In summary, the freezing temperature range is excessively wide and settings are inconsistent. This indicates that the scientific principle by which freezing transforms starch noodle texture from sticky paste to non-sticky, tough retrograded gel has not been fully clarified. Insufficient knowledge of the mechanism for optimizing freezing temperature to improve texture has seriously hindered understanding of temperature-mediated gel texture transformation in starch noodles. This knowledge gap leads to empirical process modifications characterized by blind adjustments and suboptimal results. The persistent issue of starch matrix adhesion continues to impair production efficiency and economic feasibility.

Because starch noodles are high-concentration starch gel (starch concentration > 35%) [3,5], controlling freezing temperature and duration is a crucial scientific issue both in practical application and for revealing the mechanistic relationship between freezing parameters and starch gel network evolution. The retrogradation of low-concentration starch gel (<20%) is dominated by amylose during short-term retrogradation; subsequently, interactions between starch chains strengthen during long-term retrogradation, while binding affinity between starch chains and water molecules weakens, leading to hardening [10], gel volume shrinkage, and water separation [11]. This property is utilized in sauces/dairy products to maintain liquidity [12]. However, high-concentration gels (>20%) contain more ordered molecular structures due to lower water content, higher density of starch molecules, and retained short-range ordered structure (e.g., single and double helices) [13], resulting in springiness loss and pulverization. This makes them suitable for texture setting in gel candies/plant-based foods [14]. To date, the extensive literature has focused on freezing temperature effects on low-concentration starch gels (<20% starch) [15,16], and retrogradation near T_g_’ promotes the formation of crystal nuclei [17,18]. The glass transition temperature (T_g_’) is a key kinetic parameter for multiscale food stability. When storage temperature is below T_g_’, molecular migration rates decrease by orders of magnitude, effectively inhibiting water migration, starch retrogradation, and oxidation reactions, thereby extending texture life of frozen/low-moisture foods. Modern food design recognizes T_g_’ regulation as a core strategy to extend shelf life and enhance functionality [19]. When freezing temperature is lower than T_g_’ [20,21,22], lower retrogradation results in a more viscous, softer, unformed, and fragile texture in starch noodles [1]. Conversely, soft bread and cake can delay staling and moisture loss by replacing 10–20% wheat flour with specific starches, maintaining lower retrogradation levels and extending shelf life by 3–5 days [23].

In summary, reasonable control of freezing temperature and time is the key to potato noodle processing, and the texture forming progress of high-concentrated starch gels frozen at below and above T_g_’ should be addressed. Therefore, this study aims to investigate the effects of different freezing temperatures (specifically T_5_ (−18 °C) < T_4_ (−12 °C) < T_3_ (−6 °C) < T_g_’ < T_1_ (freezing point)) on texture formation and surface compactness of PSN, clarifying the key role of T_g_’ in this process. These findings will provide theoretical evidence and practical insights for optimizing freezing processes of starch gel-based foods through T_g_’ temperature control.

## 2. Results and Discussion

### 2.1. Determination of Freezing Curve and Glass Transition Temperature

**Figure 1:**
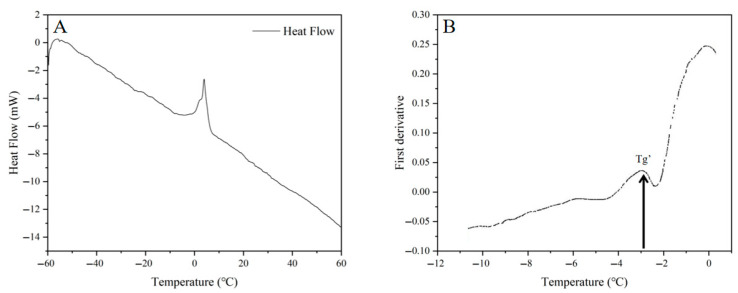
Heat flow curve (**A**) and first derivative of the heat flow curve from −11 °C to 0 °C (**B**) of potato starch gels.

The freezing curve reflects the variation in material temperature over time during the freezing process [24]. As shown in the freezing curve (Figure S1), the core temperature of the PSN reached freezing point at −1 °C. The supercooling point dropped to −4.5 °C, requiring 4 min to reach it. At this point, the liquid water begins to freeze, forming a crystal nucleus and releasing heat, causing a slight temperature increase. Subsequently, as freezing progresses, the temperature of the gel center decreases slowly [25]. Figure 1 shows the heat flow curve (Figure 1A) and its first derivative curve (Figure 1B) for PSN during DSC scanning (−60 °C to 60 °C). The arrow position on the first derivative curve indicates the glass transition temperature (T_g_’) of −3.1 °C [26]. It is reported that when starch gel water content exceeds 22%, T_g_’ is lower than room temperature, typically ranging between −3 °C and −10 °C [18]. Jiang et al. [27] report that as potato starch gel concentration increases (4%, 8%, 12%, and 16% (*w/w*)), freezing point temperatures decrease from −0.7 °C to −1.4 °C, while T_g_’ increases from −3.69 °C to −3.23 °C. In this study, the starch concentration was 35%, significantly higher than in Jiang et al. [27]. Consequently, the detected T_g_’ was higher than their values. Li et al. [28] studied the relationship between glass transition properties of frozen wheat dough and shiitake mushroom polysaccharide molecular weight. Their results showed that adding 2% high-MW (20 kDa) polysaccharide increased the dough glass transition temperature from −29.93 °C to −20.59 °C. Baek et al. [29] compared the effects of sugars/sugar alcohols (ribose, xylose, glucose, fructose, mannose, sucrose, maltose, isomaltose, trehalose, xylitol, mannitol, and sorbitol) on the thermal behavior and storage stability of corn starch gel (40% starch; starch/sugar = 10:1 or 10:3 *w*/*w*). They found that melting temperature and T_g_’ decrease with increasing sugar molar concentration.

### 2.2. Texture Characteristics of PSN

**Figure 2:**
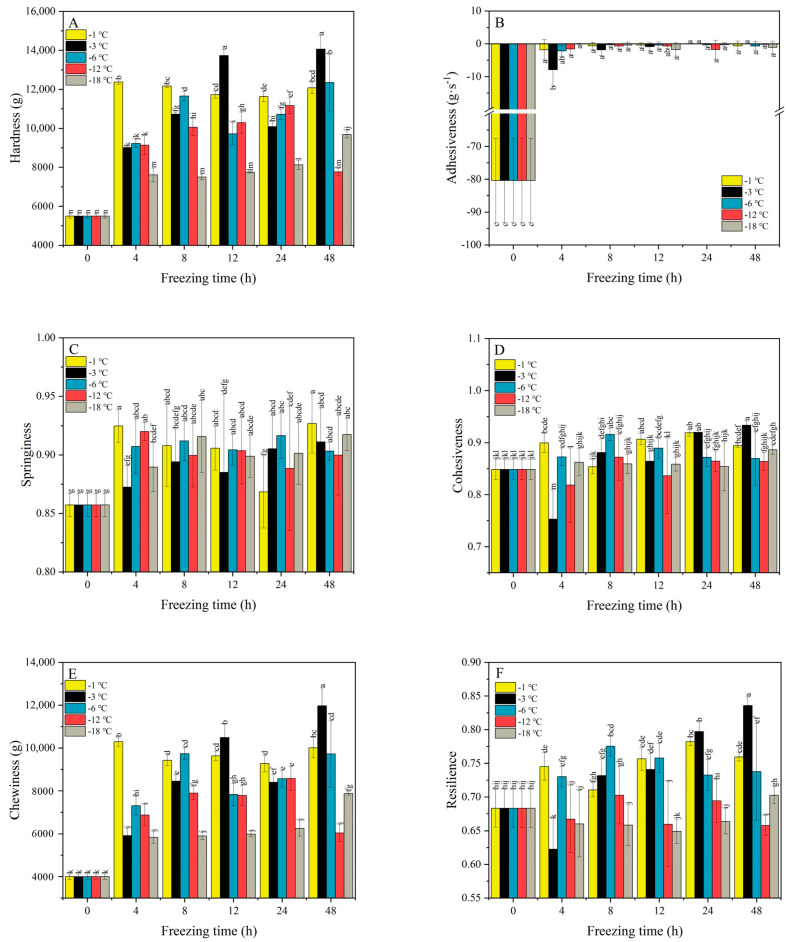
Effect of different freezing temperatures and times on textural properties of PSN (Textural properties: (**A**) Hardness, (**B**) Adhesiveness, (**C**) Springiness, (**D**) Cohesiveness, (**E**) Chewiness, (**F**) Resilience). The letters in the figure represent the differences between different groups.

Hardness is defined as the force required to achieve a specific deformation [30]. As can be seen from Figure 2A, the hardness first reaches a steady state at −1 °C after 4 h, and then the upward trend gradually slows down. This is due to the appearance of a solid, delicate, and uniform gel network structure in the microstructure of the gel at this temperature [25,31,32]. The hardness at 3 °C and −6 °C increased from 0 h to 12 h, with no ice crystal formation during this process. Starch retrogradation formed a uniform gel structure. During the first rising stage, the hardness of PSN also reached its maximum value (13,734.096 g) at −3 °C for 12 h. Hardness decreased from 12 h to 24 h, likely due to ice crystal formation during water freezing, which disrupted the three-dimensional gel network [33]. However, between 24 h and 48 h, starch transitioned into a glassy state, increasing hardness [34]. Notably, long-term storage (>48 h) disrupts texture due to ice crystal growth [35], causing surface dehydration, void formation, brittle texture, and fracture [16]. Compared with −1, −3, and −6 °C, the hardness of −12 °C and −18 °C shows an overall upward trend, but the growth rate is significantly slower than −1, −3, and −6 °C (Figure 2A). This may occur when temperatures slightly higher than the glass transition temperature accelerate the retrogradation of starch gel and starch foods, and increase hardness and chewiness [36,37]. Simultaneously, the gel may enter a glassy state at extremely low temperatures, inhibiting retrogradation [27].

Adhesiveness is used to characterize the degree of cohesion in the internal structure of a sample [38]. The initial adhesiveness value of PSN was 80.373 g·s^−1^. After freezing at different temperatures, the adhesiveness significantly decreased with time (from 80.373 g·s^−1^ to 0 g·s^−1^). This indicates that freezing treatment can significantly reduce the adhesiveness of the PSN’s surface. The adhesiveness of PSN after 4 h of freezing treatment at −12 °C and −18 °C (0 g·s^−1^ and 0.097 g·s^−1^, respectively) was close to 0 g·s^−1^. As the freezing time increased, the adhesiveness of PSN also tended to zero at −1, −3, and −6 °C. However, the trend of adhesiveness toward 0 at −12 °C and −18 °C was greater than that at −1, −3, and −6 °C. This may result from lower temperatures increasing water molecule mobility post thaw, reducing gel elasticity and smoothing the surface [21].

Springiness refers to the height ratio of a compressed deformed sample that returns to its pre-deformation condition after removing the deformation force [39]. Figure 2C shows springiness at −1, −3, and −6 °C increasing continuously from 0 to 12 h, peaking at 0.894 (−3 °C, 8 h), followed by a gradual decline. However, the temperatures of −12 °C and −18 °C increased from 0 h to 12 h. The maximum value is 0.915 (−18 °C, 8 h). Then, there was a continuous and slow downward trend. There was a small increase after 24 h. This indicated that within a certain range of freezing treatment, the springiness of PSN could be significantly improved. Previous studies suggest an optimal freezing range of −10 °C to −3 °C, where freezing is fastest. Temperatures outside this range reduce springiness and hardness [40].

Cohesiveness reflects intermolecular attraction strength and structural recovery after deformation [41]. As shown in Figure 2D, the cohesiveness fluctuates frequently, with the maximum value at −3 °C for 48 h (0.933) and the minimum value at −3 °C for 4 h (0.753). This may be because the potato starch gel has a faster recovery rate at −3 °C [25], intensifying competition between retrogradation and ice crystal formation. However, at −12 °C and −18 °C, cohesiveness stabilized and increased. Seetapan et al. [16] found that compared with a slow freezing rate, a higher freezing rate can better maintain the texture characteristics of starch gel samples with a total starch concentration of about 40%, because the water may undergo vitrification (or glassy ice formation) without any ice crystal formation.

Chewiness refers to the energy required to chew a solid sample into a stable state when swallowed [42], and is calculated from hardness, cohesiveness, and springiness. Chewiness exhibited similar trends with hardness at each freezing temperature, i.e., increasing initially, then decreasing within 24 h, with the maximum value occurring at −3 °C after 48 h (11,971.059 g). Values remained higher after 48 h, potentially due to glassy gel formation.

Resilience refers to the degree to which a sample can be restored to its original state after deformation under the same speed and pressure conditions [43]. Figure 2F shows resilience generally rising, then declining. Samples at −3 °C exhibited the greatest recovery, peaking at 0.835 units (−3 °C, 48 h) with a minimum of 0.622 units (−3 °C, 4 h). With the decline in freezing temperature (−6, −12, and −18 °C), the trend of first rising and then falling becomes stable. 

### 2.3. Principal Coordinate Analysis (PCoA) and Cluster Analysis

**Figure 3:**
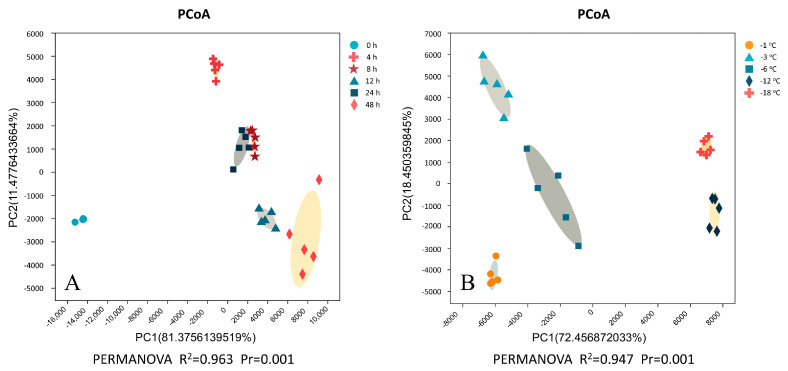
Principal coordinate analysis and PERMANOVA analysis based on textural data of PSN for different groups: freezing times (**A**) and freezing temperatures (**B**).

To determine the decisive factors affecting texture dissimilarity among PSN samples, we evaluated the relative contributions of freezing time and temperature using six texture parameters (hardness, adhesiveness, springiness, cohesiveness, chewiness, resilience) via principal coordinate analysis (PCoA) and permutational multivariate analysis of variance (PERMANOVA) (Figure 3A,B). The first principal component primarily represents adhesiveness and springiness, whereas the second represents cohesiveness and resilience. As shown in Figure 3A, when freezing time was used as a variable to evaluate the similarity between PSN, samples were divided into four clusters: Cluster 1 (0 h), Cluster 2 (4 h), Cluster 3 (8 h and 24 h), and Cluster 4 (12 h and 48 h). This highlights significant intergroup differences in freezing time. Among them, PSN samples frozen for 8 h and 24 h, and 12 h and 48 h were clustered together, indicating that the texture of these treatments was similar. The texture of PSN samples frozen at 0 h and 4 h showed significant differences compared to other samples (*p* = 0.001). When freezing temperature was used as the variable, samples were separated into four groups, namely, Group 1 (−1 °C), Group 2 (−3 °C), Group 3 (−6 °C), and Group 4 (−12 °C and −18 °C), with significant differences (*p* = 0.001), confirming distinct textures across temperature groups. Notably, the R^2^ value for time (R^2^ = 0.963) exceeded that for temperature (R^2^ = 0.947), indicating freezing time contributes more to PSN texture differences.

**Figure 4:**
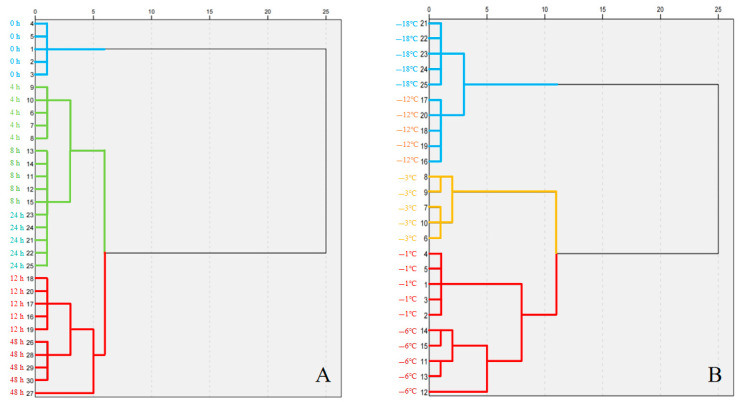
Dendrogram generated with freezing times (**A**) and freezing temperatures (**B**) based on hierarchical cluster analysis. (Different colors represent different cluster: blue represents cluster 1, green represents cluster 2, and red represents cluster 3).

The hierarchical clustering analysis divides different clusters based on hardness, adhesiveness, springiness, cohesiveness, chewiness, and resilience. From the HCA plot in Figure 4A, Cluster 1 (blue cluster) represents the 0 h group; Cluster 2 (green cluster) includes the 4 h, 8 h, and 24 h groups; and Cluster 3 (red cluster) includes the 12 h and 48 h groups. These results are consistent with PCoA, emphasizing the quality changes of samples at different time points affected by starch recovery and ice crystal growth. From the HCA plot in Figure 4B, Cluster 1 (blue cluster) represents the −12 °C and −18 °C groups, while Cluster 2 (green cluster) includes the −3 °C groups, and Cluster 3 (red cluster) includes the −1 and −6 °C groups. This consistency with PCoA emphasizes texture differences between the −3 °C and adjacent groups. The results indicate that the optimal solidification temperature range for starch is between 10 °C and −3 °C. When the setting temperature of the same setting time is higher or lower than the temperature range, the physical properties of starch gel will change [40]. This HCA grouping confirms the texture features of PSN.

### 2.4. Enzymatic Hydrolysis and SEM

**Figure 5:**
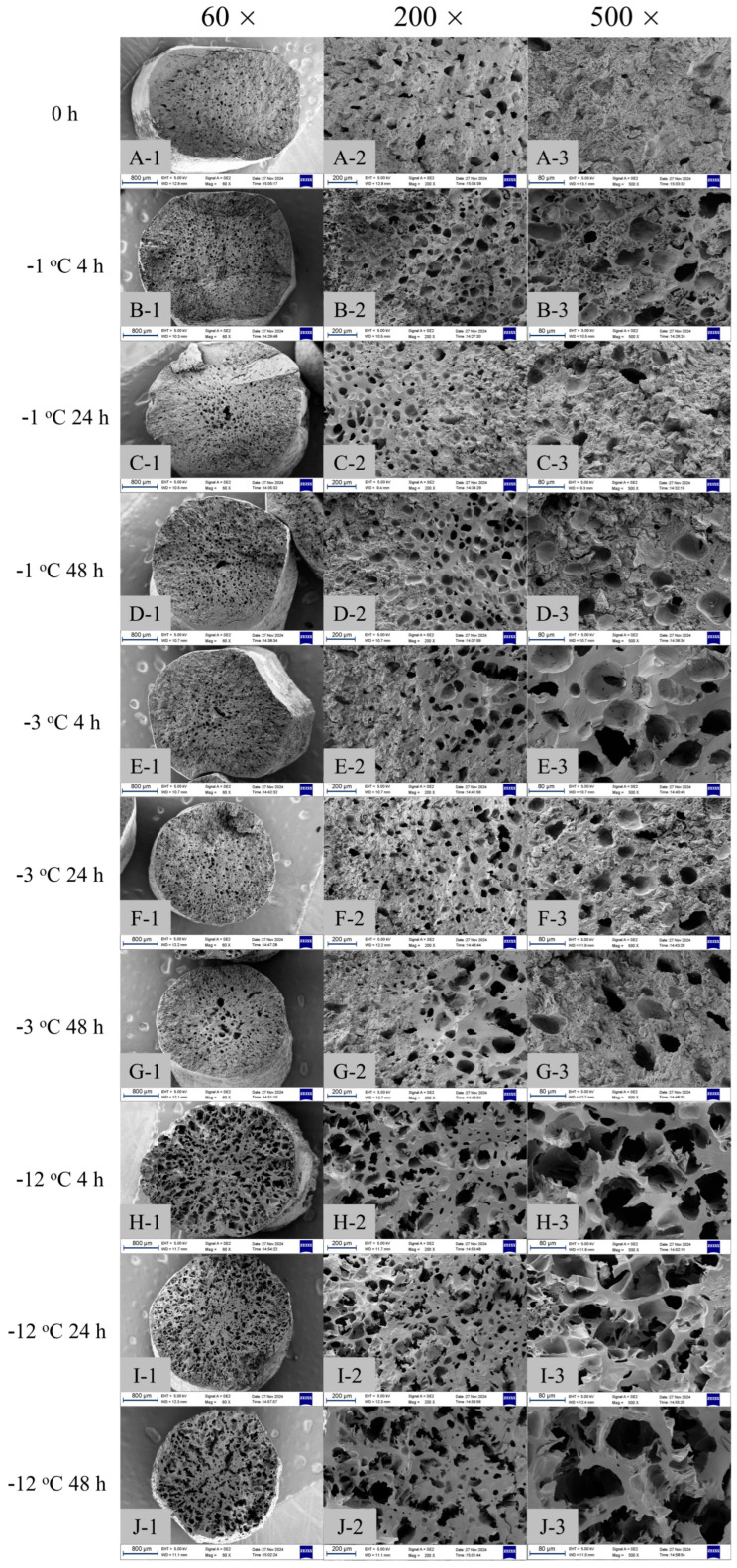
SEM images of unfrozen PSN (0 h) and frozen PSN sections at different freezing temperatures and times (Three groups of magnification (60×, 200×, 500×) freezing temperature time grouping: (**A**) 0 h, (**B**) −1 °C 4 h, (**C**) −1 °C 24 h, (**D**) −1 °C 48 h, (**E**) −3 °C 4 h, (**F**) −3 °C 24 h, (**G**) −3 °C 48 h, (**H**) −12 °C 4 h, (**I**) −12 °C 24 h, (**J**) −12 °C 48 h).

According to the results of PCoA and cluster analysis, samples at −6 °C and below had a similar texture. Therefore, PSN samples frozen at −1, −3, and −12 °C were selected as representatives to observe the cross-section morphological characteristics. The starch gel before freezing presented (Figure 5A) a typical honeycomb-like gel structure after gelatinization. These loose pore structures provide the springiness and soft texture for starch noodles [44]. However, it was accompanied by high adhesiveness. After freezing for a certain time, a fissured texture appeared on the cross-section of the PSN. The results showed that starch retrogradation made the PSN gel structure compact and able to resist boiling water pressure. The typical structure of PSN is formed. This explained the rapid decline in adhesiveness after freezing in terms of microstructure.

At −1 °C (Figure 5B–D), due to being at the freezing point temperature, most of the water molecules close to the freezing point began to form some ice crystals, which put pressure on the structure of starch molecules and may have caused small cracks in some cases. At this temperature, the molecular chains can be partially crosslinked to form a network. However, because the retrogradation process of starch is inhibited due to the temperature approaching the freezing point, there is less opportunity for the rearrangement of molecules and the reformation of hydrogen bonds, which makes the physical properties of the gel remain stable [45], but it did not achieve the best retrogradation effect. It can be seen from the figure that the structure formed by 24 h (Figure 5C) of freezing was stronger than that formed by 4 h (Figure 5B) and 48 h (Figure 5D) of freezing, probably because the 24 h starch gel had the largest retrogradation degree. This also explained why the cohesiveness after 24 h of freezing was 0.92 g, which was significantly higher than that after 4 h of freezing (0.9 g) and 48 h of freezing (0.89). In addition, it can be clearly seen from Figure 5C-1 and D-1 that a large hole appeared at the center of the PSN section and the honeycomb structure became more blurred as it approached the edge of the section. This was because when the PSN were stored at a humidity lower than the refrigerator (RH 65.59% ± 0.62%), there was a moisture gradient between the PSN’s surface and the freezing environment. In this case, the moisture in the PSN migrated from the interior to the surface, resulting in a decrease in moisture in the PSN during the initial freezing stage. On the other hand, the crosslinking degree of the macromolecular network structure in the PSN increases with the prolongation of freezing time. As a result, the transfer of water from the inside to the surface is inhibited. The transfer speed is lower than the diffusion speed of water on the PSN’s surface [46,47].

At −3 °C (Figure 5E–G), almost reaching the glass transition temperature, the water molecules in the gel were frozen. The thermal motion of starch molecules is relatively low, but it does not necessarily mean complete stillness [48]. The flexibility and fluidity of molecular chains have decreased, mainly manifested as vibration and small displacement [49]. At this time, hydrogen bond and other non-covalent interactions become more important, and the intermolecular interaction force increases, creating a more ordered structure (e.g., double helix, short-range molecular order), which helps to maintain the structural stability of gel [50]. This freezing phenomenon also led to changes in the mechanical properties of water molecules, especially affecting the hardness and resilience of the gel. It can be seen from the figure that the pore distribution formed by 24 h (Figure 5F) of freezing was more regular than that of 4 h (Figure 5E) and 48 h (Figure 5G) of freezing, which may have been because the 24 h starch gel had the largest retrogradation degree. This also explained why the cohesiveness value after 24 h of freezing was the highest, at 0.92 g, significantly higher than that after 4 h of freezing (0.75 g). At −12 °C (Figure 5H–J), due to the extremely low freezing temperature, the water contained in the gel formed ice nuclei at multiple points inside, which grew with the freezing time, destroying the structure of the gel that should have been continuously regenerated. As can be seen from the figure, the 24 h (Figure 5I) freezing had more fissured continuous structures than the 4 h (Figure 5H) and 48 h (Figure 5J) freezing, probably because the 24 h starch gel had the greatest degree of retrogradation. This also explained why freezing for 24 h had the highest cohesiveness value of 0.86, significantly higher than freezing for 4 h (0.82).

**Figure 6:**
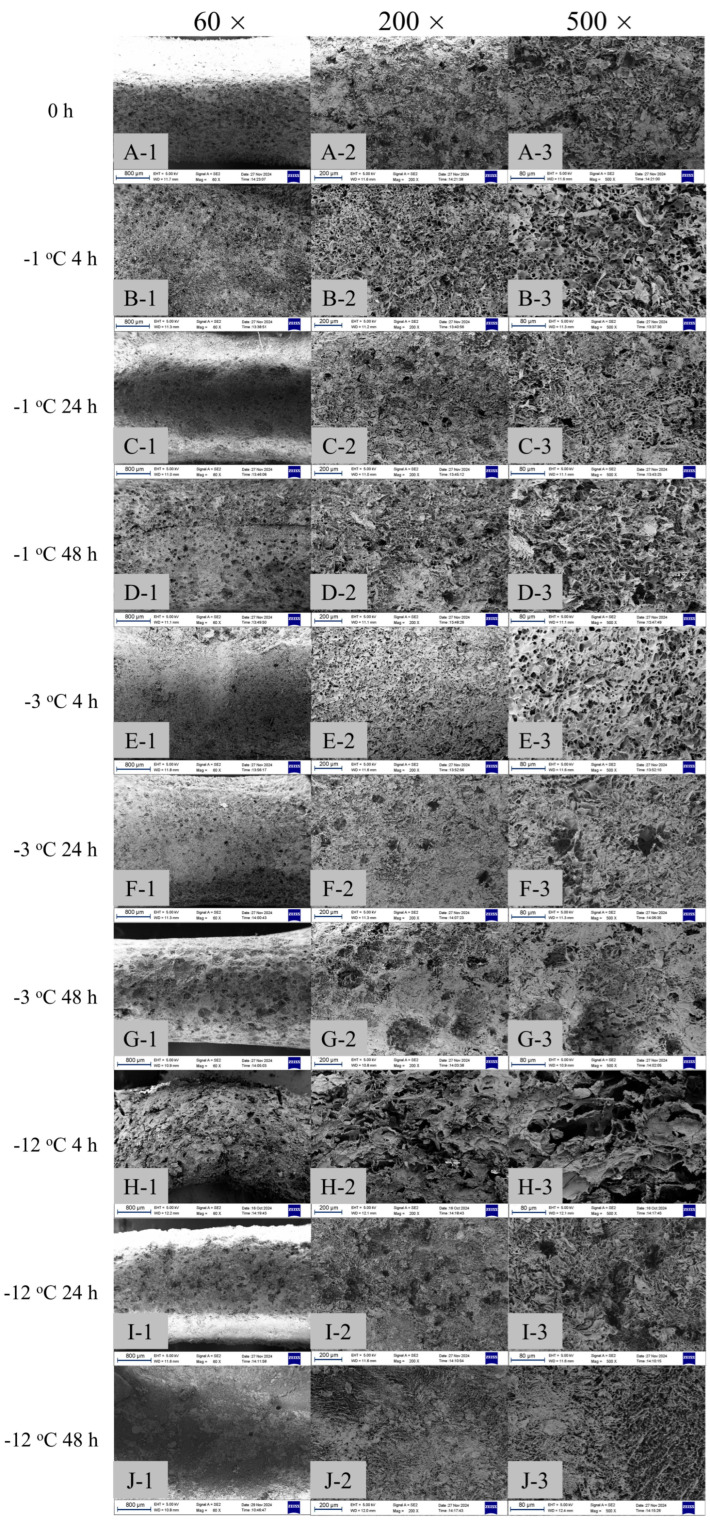
The surface SEM images of unfrozen (0 h) and frozen PSN after hydrolysis with 1% amylase for 120 min (Three groups of magnification (60×, 200×, 500×) freezing temperature time grouping: (**A**) 0 h, (**B**) −1 °C 4 h, (**C**) −1 °C 24 h, (**D**) −1 °C 48 h, (**E**) −3 °C 4 h, (**F**) −3 °C 24 h, (**G**) −3 °C 48 h, (**H**) −12 °C 4 h, (**I**) −12 °C 24 h, (**J**) −12 °C 48 h).

Figure 6 shows SEM images of PSN after 120 min enzymatic hydrolysis assessing surface firmness. In the 0 h group (Figure 6A) without freezing time and temperature treatment and only treated with α-amylase hydrolysis, the surface of the PSN was relatively uniformly hydrolyzed, showing a number of deeper and larger pores. This could be attributed to the gel-like texture of unfrozen noodles, giving more contact sites for amylase to degrade the gel to smaller fragments.

When frozen for 4 h, PSN samples stored at −1 °C (Figure 6B) and −3 °C (Figure 6E) had similar honeycomb surfaces, while samples frozen at −12 °C (Figure 6H) had cracked structures. The reason might be that the lower freezing temperature promoted the water inside the PSN to form ice crystals faster. The growth of ice crystals had a certain extrusion and destruction effect on the starch network structure of PSN, which led to the structural changes of the surface occurring more easily in the subsequent enzymatic hydrolysis process. When the freezing time reached 24 h, irregular pore structures appeared on the surface of PSN at −1 °C (Figure 6C) and −3 °C (Figure 6F), and the surface structure was more disordered at −1 °C compared to −3 °C. This might be because when the temperature dropped to −3 °C, the starch molecular chains underwent cooling, and some molecules would cross the energy barrier, forming temporary hydrogen bonds. As the temperature decreased, the phenomenon of retrogradation might be suppressed, but small-scale hydrogen bonding could lead to local structural stability. At −12 °C for 24 h (Figure 6I), the surface of the PSN almost completely lost its original smooth structure, showing a serious state of fragmentation and spalling. This showed that the structural damage of frozen PSN for 24 h became more and more serious with the decrease in temperature, which was because the ice crystals had more time to grow and gather, causing more serious damage to the starch network structure of PSN, resulting in serious damage to the surface structure in the process of enzymatic hydrolysis. Xu et al. [21] find that during refrigeration, the onset of ice crystal expansion destroys the short-range ordered structure of starch, resulting in an increase in pore size and structural relaxation of the submicroscopic structure, making the entire surface of PSN appear as irregular macropores and ruptures.

When the freezing time reached 48 h, the degree of damage on the surface of the PSN was further aggravated. At −1 °C for 48 h (Figure 6D), most areas were broken and peeled. At −3 °C for 48 h (Figure 6G), the PSN had basically lost their original shape, and the surface showed collapse and fragmentation, and the original structure of the PSN could hardly be recognized. At −12 °C for 48 h (Figure 6J), the PSN showed a uniform but slightly hydrolyzed surface morphology, indicating a tighter and firmer surface possibly squeezed by internal ice.

## 3. Conclusions

This study provides a detailed analysis of the impact of freezing temperature on the texture formation of PSN. The results demonstrate that the freezing conditions significantly influence the mechanical and microstructural properties of PSN, with the most desirable texture being achieved when PSN are frozen at −3 °C for 24 h. Freezing below T_g_’ (at −6, −12, and −18 °C) resulted in a porous structure disrupted by the growth and extrusion of ice crystals and lower springiness, while temperatures around the freezing point (−1 °C) and T_g_’ (−3 °C) produced noodles with better texture, tighter surface, and continuous solid cross-sections. These improvements are attributed to the starch retrogradation, reduced ice crystal formation, and higher mobility of starch chains at these temperatures. This study highlights the importance of T_g_’ in the texture formation of starch gel during freezing and provides valuable insights for optimizing freezing processes in starch-based food products. Further research should focus on the mechanisms behind the texture changes of starch gels at temperatures below and above T_g_’ from the molecular view.

## 4. Materials and Methods

### 4.1. Materials

Potato starch: S11004, Shanghai Yuanye Bio-Technology Co., Ltd. (Shanghai, China), containing 17.14% moisture, 0.21% ash, 0.15% protein, 0.36% lipid, and 34.99% amylose. α-Amylase (*Bacillus subtilis*): S10002, Shanghai Yuanye Technology Co., Ltd. (Shanghai, China), enzyme activity 4000 u/g.

### 4.2. Methodology

#### 4.2.1. Preparation of PSN

Binder potato starch (20 g) was dispersed in 120 mL of water and heated in a boiling water bath for 2 min with continuous stirring to yield a totally gelatinized paste. The resultant paste was mixed with 200 g of potato starch and kneaded into a uniform and smooth starch dough. The kneaded smooth dough was placed in the artificial extrusion equipment as to apply uniform pressure on the starch powder and squeeze it into the boiling water pot (the distance between the artificial extrusion equipment and the pot should be kept at 30 cm). After being cooked for 1 min, noodles were transferred into cold water for cooling for 5 min and dried at room temperature for 2 h.

#### 4.2.2. Measurement of Freezing Point Temperature of PSN

The sensor of the contact thermometer (DT1310, Shenzhen Lihua Instrument & Tools Co., Ltd., Shenzhen, China) was inserted into the starch gel center, and the gel was placed in a cryogenic refrigerator (BCD-561WLHSS14W9U1, Haier Group, Shandong, China) at −24 °C for quick freezing. The core temperature of the gel was recorded every 2 min. The freezing curve was drawn with time as the abscissa and the gel core temperature as the ordinate [25].

#### 4.2.3. Measurement of Glass Transition Temperature of PSN

The glass transition temperature of the potato starch gel sample was measured using a differential scanning calorimeter (DSC, DSC8000, PerkinElmer, Shelton, CT, USA). The sample powder (approximately 3 mg) was accurately weighed into a 40 μL DSC aluminum crucible, and distilled water was added to achieve a starch-to-water ratio of 1:3 (*w*/*v*). Prior to measurement, the aluminum crucible was sealed and equilibrated overnight at room temperature. An empty aluminum crucible was used as a reference. The sample was scanned across the temperature range of −60 °C to 60 °C with a heating rate of 5 °C/min. The thermograms obtained from the heating cycle were used to determine the glass transition temperature (T_g_’) using Universal Analysis software (Universal Analysis 2000) of TA Instruments [51].

### 4.3. Freezing of PSN

PSN were frozen in six freezing temperature gradients, T_5_ (−18 °C) < T_4_ (−12 °C), T_3_ (−6 °C) < T_g_’ (glass transition temperature) < T_1_ (freezing point temperature) for 48 h, and samples were taken at 0, 4, 8, 12, 24, and 48 h for subsequent experiments. Blank samples were not subjected to freezing treatment and were recorded as 0 h.

#### 4.3.1. Textural Properties

A texture analyzer (TA-XT2I texture analyzer, Stable Micro Systems, Godalming, UK) was used to determine the parameters. The HDP/PFS probe was selected, and five PSN with no cracking, no bending, uniform thickness, and the same length were selected. Samples were thawed for 10 min. Subsequently, the HDP/PFS probe was used for TPA testing. During testing, surface moisture was removed by blotting with absorbent paper, and five PSN strips were placed on the test bench in parallel. This was performed at least 20 times in parallel experiments on each sample. The result was processed by removing the maximum and minimum values and averaging them. The parameters were set as follows: measurement mode: compression mode; speed before test: 1.00 mm/s; operating speed: 0.5 mm/s; speed after test: 10.00 mm/s; target mode: flexibility; flexibility: 40%; trigger type: automatic; trigger force: 3.0 g [52,53,54].

#### 4.3.2. Enzymatic Hydrolysis and Scanning Electron Microscope (SEM)

The surface microstructure of PSN before and after enzymatic hydrolysis for 120 min was observed using an SEM (SUPRATM55, Carl Zeiss AG, Oberkochen, Germany). An enzymatic solution with a pH value of 6.0 and a dry matter mass of 1% was prepared. The enzymatic hydrolysis temperature was maintained at 60 °C, and samples were cut to 2 cm, placed in the enzymatic hydrolysis solution, and subjected to continuous stirring at 150 r/min. Enzymatic hydrolysis was carried out for 120 min [55]. Subsequently, samples were removed for adhesive fixation, and the surface and cross-section of the samples were gold-coated using the sputtering coating method. Scanning electron microscopy was performed at an acceleration voltage of 15 kV, and photographs were taken; the PSN surface images were scanned at a magnification of ×50 [56].

### 4.4. Statistical Analysis

Data were processed in WPS office (Kingsoft, WPS Office 12.1.0.21915, Beijing, China); statistical analysis and cluster analysis (intergroup connection method, squared Euclidean distance) in this study were both based on IMB SPSS Statistics 27 Free Edition (SPSS, Chicago, IL, USA). Principal component analysis (Euclidean algorithm) was conducted on the Majorbio platform (https://www.majorbio.com/, URL (accessed on 6 June 2025)), and plotted data were processed by Origin 2021 (Origin-Lab, Inc., Northampton, MA, USA). The significant differences were checked by one-way ANOVA (Duncan’s test), and the value of *p* < 0.05 was deemed statistically significant. All experiments were replicated five times and data were presented as mean ± standard deviation.

## Data Availability

Data are contained within the article.

## Supplementary Materials

The following supporting information can be downloaded at https://www.mdpi.com/article/10.3390/gels11080639/s1: Figure S1: Temperature change in the center of PSN.

## Abbreviations

The following abbreviations are used in this manuscript:

PSN	Potato starch noodles
PCoA	Principal coordinate analysis
SEM	Scanning electron microscope
