# Supplementary figures and images for: Temperature-Mediated Gel Texture Transformation in Starch Noodles: In Respect of Glass Transition Temperature Tg’
Source: Gels. 2025 Aug 13;11(8):639. doi: 10.3390/gels11080639 (PMC12385678; doi:10.3390/gels11080639)

**Supplementary Materials:**

**Figure S1** Temperature change in the center of PSN.

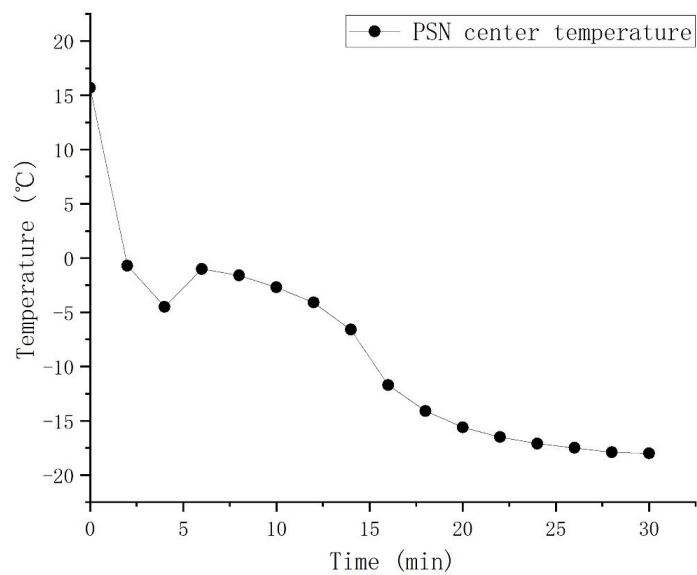

Supplement: Supplementary file 1 [file gels-11-00639-s001.zip › Supplementary Materials.pdf]
